# Extra Supplement—The Nursing Mirror

**Published:** 1889-02-16

**Authors:** 


*The Hospital,*
FEBRUARY 16, 1889.

[*Extra Supplement.*

### The Nursing Mirror.

All Contributions for this Supplement should be addressed "The Nursing Mirror," care of The Editor, 38, Old Jewry, E.C.

### En Passant.

FRIENDS IN AUSTRALIA.—Miss Maud Lobb, daughter of the late Dr. Lobb, of Hampstead, who emigrated to Australia some months ago, has been appointed matron of the Children's Hospital at Sydney. Miss Lobb and a fellow-nurse emigrated on chance, and were not a fortnight in the Colonies before they both found work. The late Sisters Cotton and Currie, of the London Hospital, have safely reached Adelaide, where they have been warmly welcomed.

NURSING AT CAIRO.—Sir Sydney and Lady Waterlow have sailed for Egypt, their object being to permanently establish a Home for Trained British Nurses at Cairo. Some nurses have already commenced work in Cairo, and their services are much appreciated, but at present they have to face many discomforts, which a permanent home will cure. The committee appointed to carry out the scheme, which has the personal approval of the Khedive, consists of Sir Evelyn Baring, Sir Sydney H. Waterlow, Bart., Dr. Greene Pacha, Dr. Milton, with Dr. Sandwith as honorary secretary.

A FRENCH NURSE.—The career of Madame Rousseil is an eminent example of national characteristics. Once a celebrated actress, this lady forsook the stage to nurse the wounded soldiers during the Franco-German War; but her enthusiasm for nursing failed when fame and picturesqueness no longer accompanied it, and in 1876 Madame Rousseil went into a convent. She did not retire quietly; she had a sudden conversion owing to the preaching of Père Didon at Notre Dame, and rumours came from the convent of the extraordinary austerities practised by the new nun. But in time the religious mania passed also, and Madame Rousseil returned to the world, and is enjoying herself in Paris.

NURSES AT BELFAST.—Perhaps a little blarney is not a bad thing; certainly nursing is improving rapidly in Ireland, and more praise is given to the nurses who perform it. At the last meeting of the Belfast Children's Hospital Committee, reference was made to the departure of Nurse Ferris, for eleven years surgical nurse to that instiitution. Pleasure was also expressed that the matron, Miss Lennox, who was ill last autumn is now quite well and back at her post. The matron of the Samaritan Hospital, Miss Morrison, also has been congratulated lately on having completed her tenth year of good service to that institution. The Belfast Samaritan Hospital now trains a lady-pupil yearly. The Belfast Society for Providing Nurses for the Sick Poor held its annual meeting on January 30th, and a satisfactory report was read. It has now over 200 names on its books.

PROVINCIAL NEWS.—The nurses of Newcastle Royal Infirmary are well looked after: they have good lectures from the medical staff; Miss Taylor, of Humshaugh, invites them two at a time to visit her and get change of air; and Mr. Hindmarsh occasionally treats them to tea in the suburbs.—The Nursing Institute in connexion with Bath Homœopathic Hospital is flourishing, and a third nurse (Miss Sellars) has been engaged.—On January 24th Miss Bradwell, matron of Louth Hospital, arranged a musical evening for her patients, which was a great success. Miss Bradwell says that a patient amused is half cured.—The matron of Princess Alice Hospital, Eastbourne, has resigned her post. —At the annual meeting of Kendall Memorial Hospital Miss Warren and the nursing staff were highly commended by the chairman.

JEWISH NURSES.—An article under this heading appears in the *Jewish Chronicle*, and strongly recommends Jewish girls to take up nursing as a remunerative employment. It seems that the Jewish nurse will have more demanded of her than is asked of the Christian nurse—she will be expected to act also as watcher over the dead. Why there are not more trained nurses of the Jewish persuasion it is hard to imagine, for, particular as some Jews are, it must necessarily be unpleasant for them to be attended by Christian women. Facilities are offered to Jewesses for training at many hospitals, and if the great need for nurses exists which the writer of this article states, then the remedy is entirely in the hands of the Jewish community, who should teach their girls what an opening for useful work is here awaiting them.

ANOTHER NURSES' HOME.—At the annual meeting of governors of Ashton District Infirmary, it was reported that in the month of March last the honorary medical officers recommended to the board that steps should be taken to lead to the training of nurses, not merely to fill up vacancies in the staff, but to supply the want of experienced nurses for private cases long felt in the neighbourhood. The governors took the matter into careful consideration. After many meetings of the board and of sub-committees, it was decided to build a nurses' home to the north of the present building, at a sufficient distance to secure a free passage of air, which home would give sleeping and day accommodation for the present staff of nurses. and for probationary nurses. The home will contain a recreation-room and sitting-room, and 14 bed-rooms, which will hold 20 beds. The extension has become a necessity. If any disaster should occur in the neighbourhood, the hospital would not be able to render much assistance, owing to all the beds being in use. Sometimes all the 48 beds in the four wards have been occupied, and other rooms have had to be used as additional wards, leaving little or no provision for emergencies.

AN ACCIDENT.—It is always to be regretted when an accident happens in a hospital, that is the place to repair injuries, not to procure them. A sad case which caused the death of a patient has just been investigated at the Brighton Sussex County Hospital. The deceased, named James Bowles, a blacksmith, thirty-three years of age, had been admitted to the hospital on Wednesday night suffering from erysipelas. At times all through Thursday and on Friday morning he was slightly delirious, but at other times he was rational, and neither the house surgeon nor the nurses considered that he required to be constantly watched. However, when the two nurses went to dinner on Friday in the kitchen at the end of the short corridor the head nurse asked a boy who was convalescent to go into Bowles's room, and call her at once if he wanted anything. He consented to do so, but unfortunately he did not go to the room. The door was open, but neither the boy, who was in an adjoining room, nor the nurses, who were not far off, heard any noise. The head nurse was absent about a quarter of an hour, and when she went to Bowles's room, where he had been left n bed, she was shocked to find him crouched up inside the guard, with his head on the fire. She dragged him off with his clothes still burning, and extinguished the flames, but he was so badly burned about the head and upper part of the body that he died the same evening. It is assumed that when the unfortunate man reached the fire he fainted and fell over the guard. After a close inquiry the jury decided that the occurrence was purely accidental, and that no blame attached to the nurses.

### The Pension Fund in the Provinces.

A FEW ERRORS CORRECTED.

A contemporary has re-published some correspondence anent the Pension Fund which appeared in the *Leeds Yorkshire Post*, but has strangely enough omitted one or two of the most important letters in favour of the Fund. Under these circumstances we reprint one of these omitted letters here, that at least some one should try and aid the cause of fair play. The fact that at a meeting of the committee of the Dreadnought Seamen's Hospital, it was unanimously resolved to affiliate the hospital to the National Pension Fund for Nurses, on the principle of an annual payment for each member of the staff who may pay at least an equal amount to the Fund out of her earnings as a nurse, is a practical answer to fault-finders. It was the unanimous opinion that the Fund had been conceived and organised upon the only wise basis, as it must tend to encourage habits of thrift and independence, while it secures an ample allowance for our nurses.

The following is Mr. Henry C. Burdett's letter which appeared in the *Leeds Post* of January 25th :—

My attention has been called, as the founder of this Fund, to the correspondence which has proceeded in your columns on the subject of pensions for nurses, which has engaged my attention for many years.

I am at a loss to understand the object of much of the criticism of the Fund's tables, which have been prepared by two of the best known men in the actuarial field. First, the late Mr. Cornelius Walford, and subsequently Mr. George King, were selected to advise upon a most difficult subject—difficult because it was impossible to know precisely how annuities upon the lives of nurses would in practice work out. Having a full knowledge of the miseries caused to thousands of the population at various times by the institution of friendly, building, and other societies upon an unsound basis, the actuaries were instructed, as the first consideration, to make the Fund absolutely safe, that is to say, financially sound as the Bank of England or the British Funds. These were their instructions, and all the criticism which has followed the issue of the tables goes to confirm the wisdom displayed in the choice of the actuaries, seeing that all that is urged against them is that the premiums are too high, that is, in a business sense, that the Fund is too sound and secure.

Assuming, although I am strongly advised to the contrary, that everything Mr. Fatkin has urged is true, what in effect does it amount to? Simply that the rates charged are too high; that is, that absolute safety is too costly for nurses' savings. All that a nurse or her friends will inquire, I imagine, is, "Are they unreasonably or unfairly high?" Surely not, for no critic, however hostile, has ventured to say that the Pension Fund tables are higher than the Government (Post Office) rates, they being, in fact, lower. Those who have laboured for years past to ascertain all the facts, with the view of founding a Pension Fund for Nurses upon an adequate basis, suitable to their requirements, foresaw that absolute safety meant full rates of premium, and they determined to anticipate criticism by making the Fund a mutual one, which should be managed under honorary direction, as opposed to paid directors, and which should provide that the utmost farthing which the money paid by any nurse into the Fund could be made to earn should be given back to her practically in full. In other words, if it is ascertained by experience that the rates charged, say, for a pension of £10 will justify one of £15 or £12, the nurse paying at the £10 rate will receive the £12 or £15 pension, as the case may be. In these circumstances, what motive can there be for criticisms such as you have published from Mr. Fatkin and others? These have no force or bearing on a mutual society like the National Pension Fund for Nurses.

Again, Mr. Fatkin declares that the National Pension Fund does not propose to pay more that 2 per cent. interest. As a matter of fact, we have the best reasons for hoping that we shall be able to give nurses 5 or 6 per cent. altogether in various forms. If the Pension Fund does not succeed in paying at least as much as this it will not be for want of financial knowledge or influence in the management. It is the precise truth to state that the Pension Fund commands the very quintessence of financial ability, and practically all the influence which the City of London possesses for the purchase and acquirement of approved securities. Seeing that the expenditure upon management, etc., has been, and will be, reduced to a minimum, and that the influence and ability brought to bear on the investment of money are the very best that can be procured, it must be apparent that the highest possible rate of interest obtainable with due regard to stability will be earned for and distributed among the members of the National Pension Fund for Nurses. At any rate, no other undertaking can possibly secure better rates of interest for the moneys entrusted to the managers by the members.

It may be well that I should point out to nurses themselves and to all true friends of theirs that the constitution and establishment of this Fund have been surrounded by many and serious difficulties, which it has taken some years of anxious thought and full knowledge to deal with adequately in the best interests of those it was desired to help. I have shown, I hope convincingly, that the only figures and criticisms produced by its opponents and critics are inapplicable because of the mutual character of the Fund, and they have indeed for this reason no bearing whatever on the National Pension Fund for Nurses. The great problem was to ascertain what a nurse could afford to put by as savings on a returnable scale out of her earnings. It is now generally admitted that a nurse of twenty-five in full work ought in her own interests to put by at least one-tenth, say, £2 to £2 10s., and in those institutions where she has all her washing, &c., free of expense, one-fifth, say £4 to £5* a-year, such savings to be so invested as to be available in case of marriage or change of profession. It is further held that every nurse on attaining fifty-five years of age should receive a retiring allowance of at least 10s. a week, say, £26 per annum, free of all deductions. All this the promoters of the National Pension Fund have the best reasons for hoping they will be able to accomplish, especially as an increasing disposition is being manifested by the managers of the hospitals and other institutions employing nurses to pay a portion of the premiums, say £2 per head per annum, on behalf of each member of the staff, subject to reasonable conditions. The difficulty presented to the actuary in the preparation of the tables may be illustrated by the statement that we have evidence to show that a nurse supposed to be used up at 55 years of age, when provided with an adequate pension, and freed from all anxiety and exposure, will live for 35 or 40 years longer. Indeed, I have to-day had under notice two such cases of nurses who have been pensioned by this society, one of whom is 82 and the other 85, both being in sound health, with every prospect of living for another ten years at least. Such cases among nurses would seem to be the rule, and not the exception. Thus a pension society which has been working for 20 years has not had one death among its nurse-pensioners during the whole period of its existence. With such facts before them, and the conviction that the Fund must be made financially as sound as the Bank of England, the difficulties of the actuaries in preparing the tables must be perceived by all reasonable people.

Such are the facts, and in commending them to the judgment of hard-headed Yorkshire people, whose intelligence I have good cause to hold in the highest estimation, may I ask in their hearing the anonymous and other critics and opponents of this honest endeavour to do a difficult piece of public work, in the safest and best way, two plain questions?

* It appeares from the Tables (F) issued by the Fund, that a nurse, aged 30 next birthday, may secure 10s. per week for life in case of disablement, by payment of £5 per annum.

1. What good will nurses derive from their statements and criticisms? 2. Are they prepared to absolutely guarantee nurses an adequate provision in the day of sickness and old age at a rate of premium which will in any sense be equal to the advantages offered to this deserving class of women by the Pension Fund, including its honorary management, its interest from investments, and its free donation bonuses? In any case, we have loyally done our best for the nurses, whose self-denying and devoted lives—some of which had been brought under our own immediate notice - have won our warmest sympathies, and induced us to spend and be spent in an honest endeavour to provide them with an independent maintenance when permanently overtaken by illness or incapacitated, and for the rest of their lives. We ask the support and sympathy of all true men and women for this noble undertaking, and our past experience justifies us in believing that we shall receive it abundantly, not only from Londoners, but from all classes throughout the country.

[The above letter closed the correspondence, as neither Mr. Fatkin nor the anonymous critics could answer the questions.

### Everybody's Opinion.

[*Correspondence on all subjects is invited. No communications can be entertained if the name and address of the correspondent is not given, or unless one side of the paper only be written on.*]

### A CORRECTION.

Miss E. R. Miles writes: In last week's issue of The Hospital, I see you announce my appointment as matron to the Children's Hospital, Gateshead. I thank you for your courtesy, but you have been misinformed concerning my previous appointments. It is five years since I resigned my post at the Children's Hospital, Brighton. Since that time I have been staff nurse at the Miller Hospital, Greenwich, afterwards holding the post you mention at Burnley. From that hospital I was appointed Sister of Male Surgical and Accident Wards at Swansea Hospital. I trust this explanation will not trouble you, but I shall feel obliged if you will kindly correct the announcement.

### A FAVOURITE GRIEVANCE.

It surprises me to hear so often the reiterated complaint of nurses belonging to private nursing institutions as to the differences between the fees they earn and the salaries they receive. My professional sisters, do you not consider that it costs something considerable to keep up the institution which is your home when you are not at work? Do you not think that if you received all you earn, and had to find yourselves in food, home, fire, etc., etc., you would oftentimes find it difficult to make both ends meet? And lastly, have you never read a story in which the words occur, "Friend, I do thee no wrong; didst thou not agree with me for a penny a-day?" and, having read it, does it not strike you that you also have made an agreement of your own free will, and in disparaging its terms you disparage your own wisdom and judgment? I, as once a nurse and now a superintendent, can assure you that it costs more than you think for to provide you with a home, and that superintendents do not make fortunes out of the work of their staffs, as some of you seem to think. A Superintendent.

### A LETTER FROM JAMAICA.

Miss A. E. Blake writes from Kingston Lunatic Asylum: Dear Mr. Editor,—At last I am trying to fulfil the promise I made you, to send as soon as possible "a full, true, and particular account" of my life in the tropics. To begin then with a brief description of the journey. It was a miserable foggy day (October 18th) when I became one of the passengers from Waterloo to Southampton Docks. As I remarked to two of my nurses, who were determined to see the last of me, "If anything is calculated to make one feel pleased at leaving one's country it is a London fog." I was very glad of the company of these two girls. I should have felt "a maiden all forlorn" had I come down alone, and I would not allow any of my relatives to come; I felt that the parting at the last moment would be more than we should be able to bear. In the same compartment was a hospital sister from Netley and her friends. We found from their conversation that she was going to Jamaica, and we jumped to the conclusion that she also had a Government appointment. But I found that she was on a very different errand; she was going to be married the day after we arrived to an army doctor stationed at Newcastle. We left the fog behind us long before we arrived at Southampton, and it was on a lovely day that we last looked at the white cliffs of our native land. I had a great dread of sea-sickness. It was a foregone conclusion with me that I should be a martyr to it; but whether I have to thank the article in The Hospital on that subject, or a draught composed of 20 grains each of pot. brom. and hyd. chlor. which I took at bedtime on the first three nights, or because I did not take brandy will never be known, but it is an undisputed fact that I was the best woman on board (I mean of course as a sailor). I was scarcely ill at all. Those who were ill were very badly off, for our stewardess was a new hand and was good for nothing, so the poor old steward had to attend the ladies as well as well as gentlemen.

I had heard so much of the gaities on board passenger ships, that I must confess to feeling rather disappointed. Truth to tell, things were rather tame. People said that this was due in a great measure to our being honoured by the presence of their Excellencies the Governors of Barbados and Trinidad (with the wife of the former and daughter of the latter), accompanied by their secretaries. Not but what they were very gracious and urbane, but there was a deal of etiquette about, and everyone else waited for them to take the lead in organizing amusements, &c. We had a dance or so on deck, which were distinctly failures, and one concert, for our amusement and the benefit of the widows and orphans of the sailors. There was a good deal of desultory singing and playing, and of *sub rosa* gambling (this being supposed to be prohibited). However, if we had not been so "deadly lively," if there had been more amusements it would, I fear, have made very little difference to poor me. Naturally, I found "much food for reflection." I had never before been more than a few hours' journey from my mother, home, and friends. I knew I was taking a very serious and irrevocable step. I had entered into an engagement with the Government to serve as matron of the Jamaica Lunatic Asylum for three years, unless prevented by mental or physical ill-health. And I knew that even at the expiration of that term if I wished to return to my fatherland, it would be a very difficult matter for me with no influence to hear of another appointment. There was only one other woman on board who was similarly circumstanced. She was going to join another lady in superintending a training institution, and was also bound for three years. We soon became good friends, but she is unfortunately located a great distance from me. Besides the passengers I have mentioned, there were a number of commercial men who had been across on business, officers and others who had been "on leave," girls who had been to France to school, etc. I must not forget to mention that we had ministers of at least four different creeds on board the good ship *Nile*. So that if we did not feel satisfied with one belief we could try "another way," as the cookery books say.

We had a delightful voyage as far as the weather was concerned. To sit under the awning on deck and gaze at the dark blue ocean was in itself a novel and agreeable experience. Yes, the days were most enjoyable, but the nights I shall never forget. In a few days the heat was so intense that sleep was almost an impossibility, especially to one troubled as I am with insomnia. There were four of us in our tiny cabin, although we were "first-class" passengers. We were on two shelves; I was on the top one. There would be, I suppose, a space of 18 inches from my face to the roof. How I wished myself one of the sterner sex for a time. However, as it was, I frequently left my bunk about two a.m. to take refuge in the ladies' saloon, where after a plunge in a delicious cold sea bath I sometimes managed to get another hour or two's sleep. I sometimes startled a sailor or two who were not accustomed to the nocturnal visits of people in petticoats. Nothing of any importance occurred until we came to Barbados; then we had a good deal of excitement and bustle. A guard of honour came to convey the Governor on shore. Boats came for those who had arrived at their destination, and for those who had to tranship to Trinidad, Antigua, and St. Kitts. There were also boats full of natives selling fruit, curios, and other things; and there were crowds of little nigger boys diving for coppers, who would dive under the ship and come up on the other side for a bit of silver. Most of us who were going further went to Barbados for part of the day, and enjoyed it immensely. It was a treat to feel one's feet once more on terra firma. We took a tram-car to Hastings, where, after some luncheon, we went to see a school and church. What interested me much more though were the lovely trees, ferns, and flowers, and the natives, in their picturesque attire, hawking fruit, poultry, etc. It did seem so comical to see perhaps six fowls alive squatting on a tray on the head of their owner and never trying to stir.

The next event was our arrival at Hayti. We did not land here, except to deliver and receive the mail bags at Jacmel. "Unhappy Hayti" was in a state of rebellion in its usual ridiculous way. We subsequently passed an American man-o-war going to her assistance. Jacmel reminded me of one of those toy-shop villages. At a distance it looked so new and clean and freshly-painted with pinks, and greys and reds, and there was, of course, not a chimney or any smoke anywhere, which gave it to English eyes such an air of unreality.

By this time we had begun to know and be on good terms with our fellow passengers, although I must say that I had been friendly with some of them from the commencement of the voyage, and had made one at a whist party every night; so that if I had not had to sleep in a Turkish bath I should have been sorry we were nearing our new home.

On Sunday, November 4th, at daylight then, we could see Jamaica, and anything more lovely I had never beheld. I think it must be the most beautiful of the West Indian isles. Barbados was too flat, and not sufficient wooded; Hayti looked so gloomy, but Jamaica seemed a perfect paradise, with its splendid range, "The Blue Mountains," thickly clad with tropical trees and plants. But enough of this! Is it not written in the chronicles of Froude, and a hundred other far abler pens than mine?

(*To be continued.*)

### WAGES OR CHARITY?

Nurse Storey writes:—Reading in The Hospital a Colonist's article, "A Land of Charity," I write to say that I fully endorse many of his statements. We are a charitable people. But are we a fair people? We give money by thousands to a hospital or an asylum. But do we give just wages to those whom we employ? And if we do not return fair value for the work that has been given us, do we not defraud those from whom we accept that work? Therefore, what good does our charity do us, if we rob one to give to another? Let us be just before we are generous. This practice of being bountiful with other people's money is especially noticeable in the working of so-called religious private nursing institutions. The estimable people who are at the heads of these places cloak their extortion under the name of religion, and call it charity to work their nurses to the very uttermost limits of their endurance, while they take from them nearly all their earnings to give in their own names, as their fancy or their vanity may dictate, to people who very often do not deserve it, and who at any rate have never worked for it. Call this religious? Call it honest? Absurd! They hurl scriptural texts and maxims at your head by the dozen, seemingly forgetting the maxim God himself gave to man, "The labourer is worthy of his hire." Nay, more —they seem to imagine that anyone else is far more worthy than he or she who earns it. If they are so charitably inclined, why don't they open their own purses; why don't they deny themselves. Or, if they do force us to give *such charity*, why don't they give us the credit? They tell you if you venture to demur—"If you don't like our rules you are not obliged to join us." True, in some cases. But unfortunately many of us are obliged to join them—we must live. Our necessity makes us their victims—and they are not slow to take advantage of the poverty from which many of us suffer. No, if I join an institution let it be one where there is no canting pretence; where the managers unblushingly tell me: "I know I am not offering you sufficient value for the work you have to do. I know that your necessity puts you in my power in a measure, and as I want to make as much money as I can for myself I do not scruple to tell you that if I could help it I would give you nothing at all for your services." There is an honest brutality in speeches like this that commends itself far more to my fancy than the hypocrisy of the other class, who answer the complaints of their victims with a proverb, and try to still their own conscience with a text. "The Devil can quote scripture to his purpose." And why is it that private nurses are so much in the power of these people? Why is it that these institutions thus monopolise nearly all the work that is to be had? Is it because people are still so narrow-minded and stupid as to look with suspicion upon a woman who trys to fight the battle of life alone; who will not allow herself to be cheated, but claims the right to dispose her own charity, and who rebels against imposition, whether sacred or otherwise? Why cannot English institutions follow the example of that excellent Home in Sydney, where there the nurses receive not only a fixed salary of £26 a-year with outdoor and indoor uniform, but also get the half of their ordinary fees and three-fourths of fever cases, etc.?

### NURSES AS STEWARDESSES.

A Private Nurse writes:—Your short article ("En Passant") as to nurses becoming stewardesses is very interesting. I have many times wondered that some of our large shipping companies have not tried trained nurses as stewardesses. I have heard and known of people who have been very ill indeed from sea-sickness, and there are many people both rich and poor, who would part with their friends who are taking a sea-voyage for their health, with far lighter hearts and greater confidence, could they but feel they were leaving those who are near and dear to them in the hands of a hospital-trained nurse. I myself am a young nurse, and belong to a private nursing institution, where six months' notice has to be given when leaving, or a forfeit of £50, which few nurses could afford to lose; but from my first becoming a nurse I have longed for the idea being gratified of becoming a stewardess on board a ship, and have thought many times of writing to your interesting paper for information as to where to apply. I think if a register were opened many nurses would avail themselves of the opportunity of becoming stewardesses; but of course even trained nurses could not ensure that they would not be sea-sick.

J. M. B. writes:—A nurse I know has been out as a stewardess since she had her training. She thinks that a few suggestions from her might be useful now that the subject has come up. She thinks that the stewardess ought to be a more independent person than she is. That no one but the captain, purser, or doctor ought to have the power to order her to do anything. A set of printed rules with the duties of the stewardess plainly put forward might be useful. A home for stewardesses would be a good thing too, as those who have no home have to go into lodgings when they come into port.

### CATHOLIC NURSING SISTERS.

A. B. writes: Will you allow me to express in your pages my belief that religious toleration is not confined to the army and the Middlesex Hospital? During my probation at St. Bartholomew's Hospital (1882-1885) the Roman Catholic nurses, five of whom were personally known to me, had special Sunday morning passes, in order to attend their own places of worship. I do not think any nurse was ever compelled to attend divine service against her conviction. Liberality and large-mindedness in this, as in other matters, are simply the outcome and proof of advanced culture.

### Examination Questions.

[Copies of similar questions will be gladly received by the Editor.]

The following are specimens of questions set to ambulance pupils:—

1. How many bones is the human skeleton composed of?
2. Name bones in trunk, head, and extremities.
3. Name organs contained in thorax.
4. Name organs contained in abdomen.
5. Name the principal arteries in upper extremities.
6. Name the principal arteries in lower extremities.
7. What are the various kinds of hæmorrhage (bleeding)?
8. How are they distinguished one from the other?
9. What means are adopted to stop them?
10. Name the various kinds of fracture.
11. How are they distinguished from dislocation.
12. What is done in cases of fracture?
13. Dislocations: How are they distinguished from fractures?
14. What is done in cases of dislocation?
15. Name some of the causes which produce unconsciousness.
16. How is unconsciousness tested, to see if genuine or not?
17. What is done in a (*a*) fainting fit; (*b*) appoplectic fit, and (*c*) hysterical fit?

W.H.T.

### Appointments.

Northampton General Infirmary.—We announce with much pleasure the appointment of Miss Margaret E. Winterton as superintendent of nurses at this institution. Miss Winterton entered the Nightingale Training School at St. Thomas's Hospital in January, 1881, went through all the wards as probationer, and took temporary sister's duties; in December, 1881, she was appointed night staff nurse in a male medical ward in the hospital, where she remained till August, 1882, when she was sent to Egypt as military nursing sister, on Miss Nightingale's recommendation. Miss Winterton nursed through the whole of the campaign, and the cholera epidemic of 1883, and received the Queen's medal and Egyptian star. She returned to England November, 1883, and took temporarily the post of night superintendent at the Liverpool Royal Infirmary, and left there in January, 1885, on being made sister of Arthur ward at St. Thomas's Hospital, which appointment she has just resigned.

Bristol Eye Hospital.—The recent vacancy of nurse-matron at this hospital has been supplied from twenty-four candidates by the appointment of Miss Jenkins, who has been for nearly three years night superintendent of the Glamorgan and Monmouthshire Infirmary, Cardiff. Miss Jenkins formerly held a post as ophthalmic sister in a London hospital, was trained at St. Thomas's Hospital, London, subsequently became sister-in-charge for about three years of a male medical and ophthalmic floor at St. Marylebone, London, subsequently sister-in-charge at Monsall Hospital, Manchester, and afterwards night superintendent.

### Vacancies.

The following vacancies are announced:—

**Matrons.**

Birmingham Eye Hospital, £60.
Wrexham Infirmary; £40.

**District Nurse Superintendent.**

Perth Sick Poor Nursing Society.

**Night Superintendents.**

Chelsea Infirmary, £30.
Glamorgan Infirmary, £28.

**Sisters.**

Glamorgan Infirmary, £28.
Royal Hospital for Children and Women.

**Nurses.**

North London Consumption Hospital; £23.

Newlands, Ryde.

220, Sauchiehall Street, Glasgow.

Victoria Home, Bournemouth.

Swansea Nursing Institute.

Tewkesbury Hospital (Night); £20.

Nurses' Institute, Canterbury.

Queen's Hospital, Birmingham; £20.

Holborn Union (assistant); £17.

County of Cornwall Trained Nurses' Home, Truro; Two; £25.

Royal Hospital for Diseases of the Chest, City Road, E.C.; £23.

Eston Hospital (near Middlesbrough); £25.

Bridgnorth Union; £25.

Southport Nurses' Home.

Hospital for Epilepsy. Regent's Park.

Blackheath Institute; £20.

Nursing Institution, 96, 97, 98*a*, Wimpole Street, London, W., Mr. Wilson has several vacancies for thoroughly-trained Nurses.

Nottingham and Notts Nursing Institute; Several; £25.

Wakley Convalescent Home, Longcross, Chertsey, Surrey; £20.

Kingston Nurses' Home (Hull).

Llandrindod (Radnor) Cottage Hospital.

**Midwives.**

Belfast Dispensary; £30.

Woodlands, Glasbury.

Glasgow Sick Poor Nursing Association.

**District Nurses.**

Leeds District Nurses' Home; £25.
Corbridge-on-Tyne.

**Probationers.**

Royal Infirmary, Bristol.

Bath Nurses' Home (Two).

Nurses' Home, Stoke-on-Trent.

Coldstream Cottage Hospital.

Lowestoft Hospital.

**Wardmaid.**

Andover Cottage Hospital.

### Notes and Queries.

**Queries.**

(107) *Emigration.*—To whom should I apply for information regarding nurses who wish to emigrate to Sydney?—*Nurse Jessie.*

(108) *Dictionary Wanted.*—Which is the best handy dictionary of medical terms?—*Student.*

**Answers.**

(107) *Emigration.*—Apply to Lady Samuel, 15, Courtfield Gardens, Earl's Court.

(108) *Dictionary Wanted.*—Get Hoblyn's Dictionary from Whitaker's. Price 12s. 6d.

(109) *Nurses as Stewardesses.*—We thank all our correspondents on this subject. All those who have sent particulars have had their names registered. It is impossible to answer privately all the letters received on this subject.

